# Prevalence of Amphistomes and Fasciola in large ruminants reared by smallholders in Lampung and Banten Provinces, Indonesia

**DOI:** 10.14202/vetworld.2023.2104-2109

**Published:** 2023-10-14

**Authors:** Eny Martindah, Dyah Haryuningtyas Sawitri, April Hari Wardhana, Fitrine Ekawasti

**Affiliations:** 1 Research Center for Veterinary Science, National Research and Innovation Agency, Bogor, 16911, Indonesia; 2 Indonesia Research Center for Veterinary Science, the Indonesian Agency for Agricultural Research and Development, Ministry of Agriculture, Jakarta

**Keywords:** amphistomes, Fasciola, Indonesia, large ruminants, prevalence

## Abstract

**Background and Aim:**

Parasitic diseases, including trematode invasions, result in losses to livestock in Indonesia, hindering the government’s efforts to achieve meat self-sufficiency. This study aimed to estimate the prevalence of Amphistomes and Fasciola in large ruminants reared by smallholder farmers.

**Materials and Methods:**

Fecal samples from 199 buffalo and cattle were collected from the districts of East Lampung (Lampung Province) and Lebak (Banten Province). Fecal samples were examined for the presence of trematode eggs using a sedimentation technique.

**Results:**

Parasite invasion rate was 48.2% (95% confidence interval [CI]: 41.3%–55.2%). Rate of invasion was 63.3% (95% CI: 52.7%–73.9%) in Lampung and 38.3% (95% CI: 29.6%–47.0%) in Lebak-Banten. The prevalence of multiple invasions of both Amphistomes and Fasciola was 20% in buffalo and local cattle, whereas invasion rate was 12.8% in crossbred cattle. Invasion rate of Amphistomes alone was 27.1%, and that of Fasciola was 4.5%. A higher invasion rate of Amphistomes (29.8%) occurred in crossbred animals. There were no significant differences between age groups for trematode invasion. The Chi-square test showed that the prevalence of trematode invasion in females was significantly higher than in males (51.5% and 30.0%, respectively). Amphistomes more commonly infected females than males (29.0% and 16.7%, respectively).

**Conclusion:**

All breeds were vulnerable to invasion by both trematode species and single invasions with different invasion rates. These findings contribute to determining the magnitude of the disease and provide a basis for studies on prevention and treatment of trematode invasion.

## Introduction

Parasitic diseases cause significant socioeconomic loss in cattle [1] due to weight loss, reduced milk production, low fertility rate, and death. In Indonesia, trematodes and other helminthic parasites cause losses to livestock, hindering the government’s goal of self-sufficiency in meat production [2]. Trematode invasion caused by common trematodes, such as Fasciola and Amphistomes, has a negative impact on animal health and reduces the economic value of large ruminants. Khan *et al*. [3] reported that >500 million cattle worldwide are at risk due to trematode invasion. *Fasciola* spp. is the primary causative agent of fasciolosis in Southeast Asian countries, including Indonesia. Cattle are mostly infected with the trematodes Fasciola and Amphistomes, which cause lower nutrition conversion and loss of weight and milk during ruminant grazing [4, 5]. Although infected animals might not show clinical signs, invasion lowers livestock performance, for instance, by slowing growth rate, lowering body weight, and reducing milk output [6, 7]. In Indonesia, economic loss due to fasciolosis has been studied by Munadi [8], who found that factors such as invasion rate, cattle weight, cattle age, and cattle origin significantly affect economic loss by 62.5%. However, in Indonesia, data on the epidemiology of trematodiasis in large ruminants are lacking, especially that caused by Amphistomes, because most studies have focused on Fasciola. Amphistomes and Fasciola are neglected parasites of ruminants, although they are widely distributed in tropical and subtropical areas [9]. Rolfe *et al*. [10] reported limited information on the epidemiology of paramphistomosis in cattle in Australia. Amphistomiasis and fasciolosis are parasitic diseases without any significant clinical signs and are difficult to detect, which causes farmers to disregard disease prevention and control [11]. Adult rumen flukes are generally well-tolerated. Heavy invasion with immature flukes can result in clinical disease associated with the intestinal phase of invasion [12].

Amphistomes and Fasciola require two hosts to complete their life cycle: A definitive mammalian host and an intermediate snail host [13–15]. The prevalence of trematodes in ruminants is linked to the presence of snail intermediate hosts in the area [11, 13]. Invasion occurs when animals ingest grass and water containing metacercaria [11, 14]. Nonetheless, information on the status of trematode invasions in cattle and buffaloes kept by smallholders is scarce.

This study aimed to estimate the prevalence of Amphistomes and Fasciola in large ruminants maintained by smallholder farmers in Lampung and Banten Provinces.

## Materials and Methods

### Ethical approval

All procedures performed in this study were under the ethical standards of the Animal Welfare Committee of the Indonesian Agency for Agricultural Research and Development with the Ethic registration number: Balitbangtan/BB Litvet/Rm/01/2019.

### Study period and location

**Figure-1:**
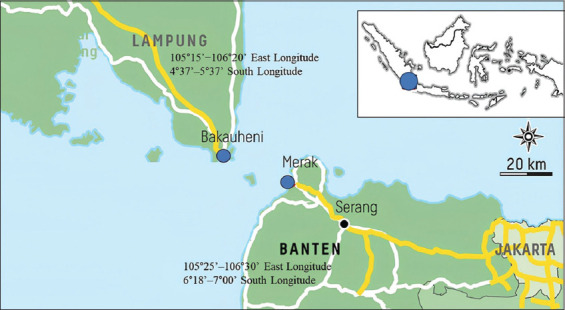
Location of fecal sample collection in Indonesia (Lampung and Banten) [Source: https;//www.magma.esdm.go.id].

A cross-sectional study was conducted on smallholder beef cattle ruminants (local cattle and crossbreed) and buffalo farms in the Regencies of Lebak (Banten Province) in March 2019 and East Lampung (Lampung Province) in October 2019. Both regencies experience a tropical climate. Geographically, East Lampung Regency is located at the position: 105°15’−106°20’ East Longitude and 4°37’–5°37’ L.S., with a distributed annual rainfall of 2000–2500 mm/year with an average air temperature of 24°C–34°C. Lebak Regency is located at 105°25’–106°30’ East Longitude and 6°18’–7°00’ L.S., the average annual rainfall reaches 2000–4000 mm and has air temperatures ranging between 24°C and 30°C (Figure-1).

### Sampling

In total, 199 fecal samples were collected from 25 farms in Lebak-Banten (n = 120) and 19 farms in East Lampung (n = 79). Buffalo and cattle fecal samples were collected from each animal, placed in a plastic bag, and sent to the laboratory. The samples were stored in a refrigerator until use. On sampling day, sex, age, breed, deworming history, and grazing management data were recorded on a sheet.

### Parasitological examination

**Figure-2:**
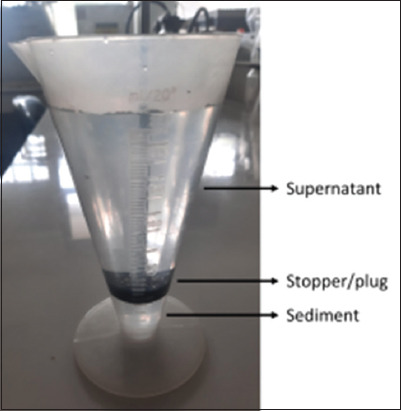
Cone tube for fecal sediment [Source: Research Center for Veterinary Science, Bogor, Indonesia].

Fecal samples were examined for trematode eggs using Boray’s modified sedimentation method [16], which is suitable for examining Amphistome and Fasciola eggs. Briefly, 3 g of fecal sample was homogenized with 17 mL water and incubated at room temperature (27°C) for 2–4 h. The fecal suspension was sequentially filtered through 341-, 200-, and 150-μm filters, and the filtrate was collected in a beaker (cone tube 250 mL). The volume of the filtrate was adjusted to 250 mL with water, and the filtrate was allowed to stand for 3 min. A stopper (plug) (5-cm diameter) was slowly inserted into the upright cone tube while applying slight pressure to remove liquid from the top (Figure-2). The process was repeated approximately 5 times until clear supernatant was removed. The fecal sediment was placed on a clean, dry Petri dish, and emulsified with 1% methylene blue. Trematode eggs (Amphistome and Fasciola) were identified based on their morphological characteristics under a stereomicroscope with 10× magnification. Amphistome eggs appeared bluish-gray, whereas Fasciola eggs appeared golden yellow and shiny [17].

### Data management and statistical analyses

Data collected during sample collection and results of fecal examination were entered and stored for analysis in a Microsoft Excel spreadsheet. An animal positive for at least one egg was considered positive for the respective trematode invasion [14]. The Chi-square test [18] was used to compare the spread of Amphistomes and Fasciola eggs between location (Lampung and Lebak-Banten), sex (male and female), and age (<2 and ≥2 years) at a confidence limit of 5%, and was computed using the following equation:

χ^2^ = Σ [(│Obs-Exp│−0.5)^2^/Ex]

Moreover, p < 0.05 was considered statistically significant.

## Results

The results of the trematode parasite invasion rate are summarized in Table-1. Trematode eggs were found in 48.2% (95% confidence interval [CI] : 41.3%–55.2%) samples. Invasion rate was 63.3% (95% CI: 52.7%-73.9%) in Lampung and 38.3% (95% CI: 29.6%–47%) in Lebak-Banten. The value Chi-square statistical calculation, at a significance level of 5%, was smaller than the Chi-square table value, suggesting that the prevalence of trematode invasion in Lampung was significantly higher than that in Lebak-Banten. In Lampung, single invasion rate of Amphistomes was 32.9% (26/79 samples), and coinvasion rate of both Amphistomes and Fasciola was 26.6%. In Lebak, the single invasion rate of Amphistomes was 23.3% (28/120), and coinvasion rate of Amphistomes and Fasciola was 10%. Single invasion rate of Fasciola in both locations was relatively low: 3.8% in Lampung and 5% in Lebak-Banten.

**Table-1:** The prevalence of Amphistomes and Fasciola eggs in ruminants (Buffalo, local cattle, and crossbreed data combined) by location, age, and sex.

Variable	No. of sample (n)	Trematode	95% CI	Percentage of invasion			

				Fasc + Amphistome	Fasciola	Amphistome	χ^2^count value
Total sample examine	199	48.2 (96/199)	(41.3%–55.2%)	16.6 (33/199)	4.5 (9/199)	27.1 (54/199)	
Location							
East Lampung (Lampung)	79	63.3 (50/79)	(52.7% –73.9%)	26.6 (21/79)	3.8 (3/79)	32.9 (26/79)	4.65**
Lebak (Banten)	120	38.3 (46/120)	(29.6%–47%)	10.0 (12/120)	5.0 (6/120)	23.3 (28/120)	
Age							
Below 2 years	40	35.0 (14/40)	(20.2%–49.8%)	5.0 (2/40)	0	30.0 (12/40)	2.89*
2 years and above	159	51.6 (82/159)	(43.8%–59.3%)	19.5 (31/159)	5.7 (9/159)	26.4 (42/159)	
Sex							
Male	30	30.0 (9/30)	(13.6%–46.4%)	10.0 (3/30)	3.3 (1/30)	16.8 (5/30)	3.88**
Female	169	51.5 (87/169)	(43.9%–59%)	17.8 (30/169)	4.7 (8/169)	29.0 (49/169)	

** χ^2^ count value > χ^2^ table value 5% = 3.84.

* χ^2^ count value < χ^2^ table value 5% = 3.84. CI: Confidence interval

The Chi-square test showed that the sex of an animal significantly affected the prevalence of trematode invasion; significantly higher in females than in males (51.5% and 30.0%, respectively, p < 0.05) (Table-1). Multiple invasions of both parasites (17.8%), as well as single invasions of Amphistomes (29.0%) and Fasciola (4.7%), were more common in females than in males.

Amphistome prevalence in terms of age was 30.0% (95% CI: 15.8%–44.2%) in younger animals (age <2 years) and 26.4% (95% CI: 19.6%–33.3%) in older animals (age ≥2 years). There were no significant differences between age groups for trematode invasion. Buffalo and local cattle had invasions of both Amphistomes and Fasciola, with an invasion rate of 20% each, whereas crossbreeds had an invasion rate of 12.8% (Table-2).

**Table-2:** Presence of Amphistomes and Fasciola eggs in fecal samples based on breed.

Variable	No. of samples (n)	Trematode invasion	Percentage of invasion		

			Amphistome and Fasciola	Amphistome	Fasciola
Breed					
Buffalo	85	51.8 (44/85)	20.0 (17/85)	28.2 (24/85)	3.5 (3/85)
Local breed	20	30.0 (6/20)	20.0 (4/20)	10.0 (2/20)	0
Cross breed	94	48.9 (46/94)	12.8 (12/94)	29.8 (28/94)	6.4 (6/94)
Total examined	199	48.2 (96/199)	16.6 (33/199)	27.1 (54/199)	4.5 (9/199)

In local cattle, the invasion rate of Amphistomes was 27.1% and that of Fasciola alone was 4.5%, but there was no single invasion of Fasciola (0%). In crossbreeds, the single invasion rate of Amphistomes was higher (29.8%) than that in local cattle.

## Discussion

Our results indicated that the overall prevalence of trematode invasion in large ruminants (48.2%) in the study areas is consistent with the rate of trematode invasion in cattle along the Progo river, Yogyakarta (50%), reported by Rinca *et al*. [2], but lower than that in Central Sulawesi (85.06%) [19], as well as in Java Island (64.83%), reported by Nurhidayah *et al*. [20]. The prevalence of multiple invasions of Amphistomes and Fasciola in Lampung (26.6%) is consistent with the findings of Hambal *et al*. [17], who reported 27% prevalence in Aceh. However, the prevalence of fasciolosis in the study areas was 3.8% in Lampung and 5% in Lebak-Banten, compared with 5.55% prevalence of bovine fascioliasis in Peninsular Malaysia [13]. The prevalence of fasciolosis was 16.50% in dairy cattle in Boyolali, Central Java, Indonesia [21]. A high prevalence of fasciolosis was reported from a smallholder beef cattle farm from a palm-cow integration system in East Kalimantan, in Bali cattle, raised extensively (65.5%) and Brahman cross cattle raised intensively (32%) [22]. These results indicate that location and management system are likely the primary determining factors for the prevalence and frequency of fasciolosis.

Mixed invasion of trematode species, such as Fasciola invasion with Amphistomes, is common in large ruminants, due to similarities in life cycles [1]. In this study, invasion of both Amphistomes and Fasciola was higher in local cattle and buffalo than in crossbreeds. Buffalo and local breed cattle are raised semi-intensively, whereas crossbreeds tend to be reared intensively. Even in intensive care, Fasciola can infect the host through feed in the form of grass-containing parasitic eggs carried by *Lymnaea* spp. Invasion can occur when cows drink water containing eggs carried by snails [14]. Invasion trematodes are related to a suitable snail habitat (snail as an intermediate host) within the rearing areas. Once the cercariae find a host, they move toward the small intestine and become miracidia, which grow and reach the host liver [21].

The overall prevalence of Amphistomes in large ruminants was 27.1% (54/199), higher than those reported from Iran at 19.5% [23], cattle farms in Germany at 5.5% [24], necropsied cows in Spain at 18.8% [25], and cattle in Turkey at 8.95% [26]. However, our value was lower than those reported from bovines in Ethiopia at 51.82% [27], and at 36.7% of bovines in good body condition [9]. These differences are likely due to variations in geographical regions and environmental conditions. Hajipour *et al*. [23] reported a significant correlation between Amphistome prevalence and season, age, breed, water source, pastureland, and grazing system in Iran. Forstmaier *et al*. [24] reported a significant association between grazing and feeding fresh grass, as well as organic farming, and trematode invasion (rumen fluke and liver fluke) in Germany.

Amphistome eggs found likely belong to the genus Paramphistomum. Paramphistomum is the most common Amphistome found in cattle and other ruminants [28]. In Indonesia, other Amphistome species, such as *Orthocoelium indonesiense*, are rarely found [29]. Although both *Fasciola hepatica* and *Fasciola gigantica* infect ruminants and humans [30], the Fasciola eggs belong to *F. gigantica*, which is the only *Fasciola* spp. found in Indonesia and other tropical countries [31, 32]. We did not identify species based on only conventional microscopy; therefore, identification was performed only at the genus level.

The results revealed trematode invasion, including single invasion of Amphistomes and mixed invasion of both parasites, was significantly higher in females than males. This result is consistent with that of Gordon *et al*. [33], who performed a study in the Philippines, but contrary to the study performed in Aceh, where male cattle were more susceptible than females to Fasciola invasion [17].

Relation to age factors indicated no significant differences between age groups for trematodiasis. This result is consistent with studies conducted in Ethiopia [27] and the Philippines [33]. A study in Ghana [34] found significantly higher Fasciola and Amphistomes in cattle than small ruminants (p < 0.05), and no significant association between the prevalence of Fasciola and age group.

## Conclusion

All breeds were vulnerable to invasion of both trematode species and single invasions with different invasion rates. Local breeds, buffaloes, and females were more susceptible to mixed invasions of Amphistomes and Fasciola, whereas crossbreeds were susceptible to Amphistomes. However, there was no single invasion of Fasciola in local cattle. For animal welfare and health, studies must examine diseases caused by Amphistomes in Indonesia. Our findings will help determine the magnitude of diseases caused by Amphistomes and provide a basis for studies on prevention and treatment.

## Authors’ Contributions

EM, DHS, AHW, and FE: Conceptualization. EM, DHS, and AHW: Sample collection. EM, FE, and AHW: Data analysis. EM, DHS, AHW, and FE: Resources. EM, DHS, and FE: Writing-review and editing. All authors have read, reviewed, and approved the final manuscript.
